# Public–private partnership in primary health care: an experience from Iran

**DOI:** 10.1017/S1463423622000561

**Published:** 2023-01-09

**Authors:** Hojatolah Gharaee, Saber Azami Aghdash, Mostafa Farahbakhsh, Majid Karamouz, Shirin Nosratnejad, Jafar Sadegh Tabrizi

**Affiliations:** 1 Department of Health Management and Economics, School of Public Health, Hamadan University of Medical Sciences, Hamadan, Iran; 2 Tabriz Health Services Management Research Center, Health Management and Safety Promotion Research Institute, Tabriz University of Medical Sciences, Tabriz, Iran; 3 Research Center of Psychiatry and Behavioral Sciences, Aging Research Institute, Tabriz University of Medical Sciences, Tabriz, Iran; 4 East Azerbaijan Province Health Center, Tabriz University of Medical Sciences, Tabriz, Iran; 5 Iranian Center of Excellence in Health Services Management, Tabriz Health Services Management Research Center, Health Management and Safety Promotion Research Institute, Department of Health Economics, Faculty of Management and Medical Informatics, Tabriz University of Medical Sciences, Tabriz, Iran

**Keywords:** East Azerbaijan Province, primary health care, public–private partnership

## Abstract

**Aim:**

The aim of this paper is to introduce the experience of applying public–private partnership (PPP) in providing primary health care (PHC) in East Azerbaijan Province (EAP), Iran.

**Background:**

Moving toward the Universal Health Coverage (UHC) involves using of all health-related resources. Certainly, one of the key strategies for achieving UHC is PPP. Since 2015, a PPP in PHC policy has begun in EAP as a major strategy for strengthening the health system and achieving UHC.

**Methods:**

In this case study, data were collected through interviews with stakeholders, document analysis, reviewing of health indexes and published studies. The data were analyzed using content analysis.

**Finding:**

PPP in PHC policy was designed and implemented in EAP with the aim of social justice, strengthening the health system and achieving UHC in the framework of health complexes (HCs). HCs provide a defined service package according to the contract. The reimbursement method is a combination of per capita, fee for services and bonus methods. Part of the payments is fixed and the other part is based on the pay for quality system and paid according to the results of monitoring and evaluation. According to the study results, the most important strength of the plan is to improve access to services, especially in marginalized areas. The main weakness is not providing infrastructures before the implementation of the plan, and the most important challenges are financial, political and organizational unsustainability and, sometimes, poor cooperation by the other organizations. The findings show that PPP in PHC in EAP is an effective strategy to provide social justice, implement family practice and achieve UHC.

## Introduction

Having a healthy and productive life with acceptable life expectancy, free of disease and disability is the prerequisite for the sustainable development, in addition to being a universal right (Motlagh *et al*., 2010). There is no doubt that one of the main countries’ strategies to achieve this is primary health care (PHC) (Starfield *et al*., 2005). In Iran, PHC has been established since 1985 in the form of health networks in different cities and villages (Barzegar and Djazayery, 1981; Shadpour, 2000; Tabrizi *et al*., 2015). Despite the achievements, there have been some challenges in recent years in providing PHC, especially in urban areas, among which the most important are transformation of infectious diseases to chronic diseases, population aging, resource unsustainability, hospital-centered health services, use of untrained physicians in managerial posts, eroded primary health centers’ building and increasing urbanization and consequently marginalized population in cities (Asadi-Lari *et al*., 2004; Moghadam *et al*., 2012; Lankarani *et al*., 2013; Vafaee-Najar *et al*., 2014). Therefore, making major reforms in management style and PHC provision in Iran, especially in urban areas, seems inevitable (Heshmati and Joulaei, 2016).

### The structure of PHC in Iran

**Figure 1.**
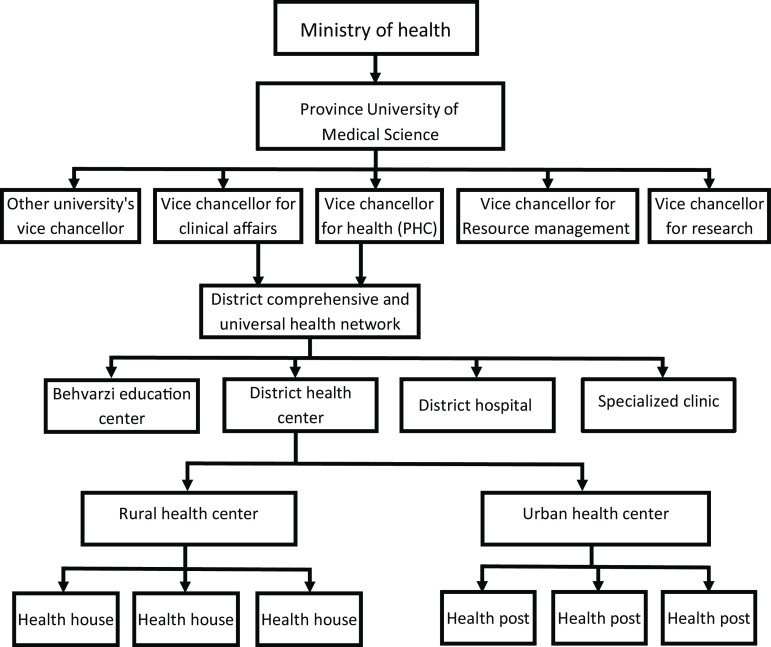
The structure of primary health care in Iran

Healthcare services in Iran are provided at three levels. The first level includes units in which the first and the widest community contact the healthcare delivery system. Service provider units at this level include health houses, health posts and rural/urban comprehensive health centers (CHC). Healthcare providers in health houses include male and female Behvarz and in health posts include family health nurses. The Behvarzes are community health workers to provide PHC in rural areas. Community members with at least primary education are recruited to the Behvarz program based on their performance in an entrance examination. Newly appointed Behvarz workers undergo 2 years of classroom and practical training before beginning work in their own local community. In addition, Behvarzes receive regular training throughout their career (Farzadfar *et al*., 2012). In urban/rural CHC, general physicians, sometimes dentists, nutritionists, psychologists and occupational and environmental health experts are usually working (Malekafzali, 2014; Sadrizadeh, 2001). The second level includes units that are able to provide more specialized healthcare services. It includes district health center, Behvarz training center, district hospitals and outpatient-specialized clinics. The set of the first- and second-level units in each district’s geographical area constitute the district health network (Shadpour, 2000; Tabrizi *et al*., 2017). The third level includes specialty clinical and educational services which are supplementary to second level. Specialty and super subspecialty hospitals and province health center and various medical schools are involved in this level. The movement of patients through these levels is carried out in the form of the referral system (Mehrdad, 2009) (Figure 1).

Today, different strategies have been used by all countries to deal with the challenges in health sector, among which public–private partnership (PPP) is one of the most effective strategies which is considered as a bilateral cooperation and win-win policy that makes both parties use their abilities to achieve common goals (Davies, 2011; Kraak *et al*., 2012). Evidence suggests that, currently, the willingness of governments to engage the private sector in health system has increased (Anderson, 2012; Mahoney *et al*., 2009; Zheng *et al*., 2008).

One of the areas taking seriously into account applying the PPP’s potentials is Universal Health Coverage (UHC). UHC is the third goal of sustainable development goals that most countries are seeking to achieve by 2030 (Iyer *et al*., 2016). The UHC’s goal is to maximize health outcomes through a full coverage of the population and to develop a comprehensive quality service package that is geographically accessible and tailored to the payment ability of people (McPake and Hanson, 2016). Success in moving toward UHC requires policies, which put public and private sectors together with the aim of providing comprehensive and quality services (McPake and Hanson, 2016). Therefore, certainly one of the basic strategies for achieving UHC is PPP (Iyer *et al*., 2017).

In 1997, after the agreement of the Ministry of Health (MOH) with the Ministry of Cooperatives, with the support of senior managers of Tabriz University of Medical Sciences (TUOMS), health cooperatives plan was developed by TUOMS and implemented as a pilot in the province. Tabriz health cooperatives are known as a model of PPP, which provide PHC in the format of a defined and integrated service package to the covered population, using a controlled market model and private sector approach. The public sector paid the cost of health services through capitation and bonus based on the performance and quality of the provided services which are assessed via a continuous evaluation (Farahbakhsh, Sadeghi-Bazargani, *et al*., 2012; Nikniyaz *et al*., 2006). This plan was stopped in 2005 because of changing government and service purchase policies.

In 2015, the MOH has set an agenda for health evolution plan in PHC. The plan was developed and implemented with overall goals of active coverage of whole society, providing the comprehensive and integrated service package, and maximum financial protection of people, for achieving UHC (Tabrizi *et al*., 2016). One of the support projects of health evolution plan in PHC was PPP, based on which 20 million, especially the marginalized population in large cities, were provided through contracting with the private sector according to MOH policies. Considering the background and the prior experience of East Azerbaijan Province (EAP) in PPP, implementing this plan in this province has specific features and structures that differ from the country model. The aim of this study was to investigate different aspects of PPP plan in providing PHC in EAP based on stakeholders’ views and existing documents.

## Method

This case study was conducted in -EAP, Iran. Iran is a country located in the southwest of Asia and Middle East with an area of 1 648 195 square kilometers. Based on the 2016 census, the population of Iran was 79 926 270 (18th in the world). In 2017, Iran’s GDP was 427.7 billion dollars and according to the division of the World Bank, it is considered as one of the upper middle-income countries.

EAP with a total area of 45.491 square kilometers and 3.9 million population (Population and Housing Census in 2016) is recognized as the largest and most populated province of the northwest of Iran. This province has cold and mountainous weather, and Tabriz is the capital city of the province. About 2.809 million people, about 72%, live in cities, out of which 530 000 people (19%) of urban residents live in marginal areas.

### Data collection method

Data were collected through document review, literature review, health indexes extraction and interviews with stakeholders and experts involved in the implementation of PPP plan in providing PHC.

### Research sample

In the document review stage, documents related to the development and implementation of PPP plan in PHC in EAP, including bylaws, minutes of meeting, guidelines, recalls, contracts and other related documents, with the help of relevant experts, were collected and analyzed.

In the review stage, published articles and reports on PPP in providing PHC in EAP were reviewed too. In this part, all the literature which has been published in this area since the beginning of the plan was collected and the required information was extracted.

In the next stage, the each unit’s specified health indexes, related to 2012–2017, were extracted. In case of program which has changed over these years and in case of new program, the information that was available and recoverable from the information system was extracted and used.

In the final stage of the study, semi-structured face-to-face interviews were conducted with 14 current and former directors of vice chancellor for health; directors of district health networks; faculty members with executive and research background in the field of PPP and private health complexes (HCs) managers. The sampling method was purposive and heterogeneous. This process lasted until data saturation, at which researchers felt that by including new participants, no additional data will attain. Three experts refused to participate in the interview, two experts because of not having free time and one did not state specific reason.

### Inclusion criteria for participants

Having at least 5 years of management experience or executive activity in PHC, faculty members and researchers with a background of conducting researches in the field of PPP, having at least the bachelor’s degree in one of the medical sciences and having desire and ability to participate in the study.

### How to guide the interview sessions

A few days before each interview session, a fact sheet including the explanation about the purpose of the study, data collection methods and interview questions, designated using literature review and experts’ comments and tested via a pilot test, was sent to the interviewees. At the beginning of each session, necessary information about the purpose of the session, the method and process of its implementation and how to use the data was provided. Participants were free to leave the study if they did not agree with the process of holding sessions and how to use the results. At the beginning of each session, participants were asked to sign the informed consent form if they desired to participate in the study. All sessions were audio recorded with the consent of the participants, and notes were taken simultaneously. Interview sessions lasted between 35 and 150 minutes. Each person was interviewed once, and none of the interviews were repeated. All the interviews were conducted at the workplace of participants. Conducting the interview and taking notes both did by one person.

### Data analysis method

For data analysis, content analysis was used which is a method to identify, analyze and report existing themes (algorithms) in the text and has widespread utilization in the analysis of qualitative data. It is used when theories about the studied subject are limited (Grbich, 2012; Hsieh & Shannon, 2005; Pope, Ziebland, & Mays, 2000). The analysis of the extracted data was performed using MaxQDA10 software. In this way, the recording content of each interview was immediately transcribed, reread several times to fully understand the concepts and themes, and then the data were coded and the main themes were extracted from the primary codes. Each of the available documents was analyzed by content analysis, and data were extracted and summarized by two individuals. The results obtained from reviewing indexes and documents are presented descriptively in the Results section.

To increase the consistency and accuracy of the results, four criteria (credibility, conformability, dependability and transferability) proposed by Guba and Lincoln (1982) were used. To provide credibility and conformability criteria, immersion and review by research colleagues, applying specialists and experts’ opinions and participants’ reviews, have been used. In such a way, after completing the sessions and summing up the opinions, a summary of what the interviewees’ said was provided to them for correcting and solving the mistakes and ambiguities. To provide dependability, two individuals were employed for coding. Finally, experts’ and professionals’ opinions as well as a purposive and heterogeneous sampling were used for providing transferability.

Data were analyzed by researchers and the disagreements were referred to a third researcher.

### Ethical approval

The authors assert that all procedures contributing to this work comply with the ethical standards of the relevant national and institutional guidelines on human experimentation (Ethics Committee of the Tabriz University of Medical Science. Ethical Number: TBZMED.REC.1397.597) and with the Helsinki Declaration of 1975, as revised in 2008.

Ethical issues (including the informed consent of the participants, plagiarism, duplication, etc.) are fully respected by the authors. The confidentiality principles are respected in the information of individuals. The individuals have been assured that the results would be used only for the purposes of the study not in any other cases, and each person was allowed to leave the study at any stage without any loss.

## Results

### Background of PPP in EAP

#### Health cooperatives

After the agreements has been made between the MOH and the Ministry of Cooperatives in January 1997, in the second half of 1998, Tabriz University of Medical Sciences (TUOMS) in cooperation with General Directorate of Cooperatives of Tabriz province, established health cooperatives. Purchasing PHC services from the private sector in the form of a cooperative took place for the first time in EAP. In this plan, the urban health centers were assigned to private companies and after that were called health cooperatives. The private sector was responsible for providing PHC services that were provided earlier by public sectors, and the public sector was responsible for financing, monitoring and evaluation.

Health cooperatives covered between 9 and 17 thousand people. These centers after completing the census of covered households provide PHC and outpatient health services through periodic examinations and care of family members’ health in the framework of MOH’s guidelines. Providing services was based on a defined service package including care of children under 6 years, vaccination, nutrition monitoring, pre and post-natal care, family planning, students health care, district’s environmental health control, mental health care and health education. Health cooperatives did not receive money from service receivers for providing services defined in service package. It was the responsibility of government to reimburse for the services in the form of capitation. The amount of capitation was determined based on the evaluation’s results and the health cooperative success rate in the implementation of service package. Reimbursement was made once every 3 months, and evaluation was also conducted every 6 months by the experts of province and district health center. All current costs of health cooperatives, except for the paper forms, vaccines, some medicines and health commodities, were borne by the cooperative, and if the location were provided by the public sector, rent would be deducted from the reimbursement money. These centers received non-contractual services’ fee from the insurance companies or directly from people, according to governmental tariffs. The results of various studies that investigated the success rate of health cooperatives plan from different aspects indicate a positive impact of implementation of the plan on various aspects of PHC system (*Nikniyaz et al*., 2006; Farahbakhsh *et al*., 2007; Nikniaz *et al*., 2007; Farahbakhsh *et al*., 2011; Farahbakhsh, Sadeghi-Bazargani, *et al*., 2012; Farahbakhsh, Tabrizi, *et al*., 2012). Despite the positive results, this plan was stopped in 2005 because of changing government and changing service purchase policies due to macro-politics of the country. After this, the structure of PHC was changed to pre-health cooperatives plan (Figure 1) and was again run by the public sector. The service-providing location which rented out to the health cooperatives was returned to the district health center, and PHC services were again provided in these locations by the public sector. On the other hand, the health cooperative’s staff were recruited by public sector.

#### Health complexes

Since the beginning of 2014, after a long time, when the new government came with the slogan “Promoting people’s health” created conditions that experts who believed in PPP and were often involved in health cooperative plan were appointed again, and consequently using PPP in PHC again were discussed. Based on the study of upstream documents, world’s successful experiences, experiences gained from health cooperatives and a comprehensive analysis of the present status of public and private sectors, a preliminary plan has been developed for launching the HCs as a fundamental strategy for strengthening health system and achieving the UHC (Takian *et al*., 2015).

**Figure 2.**
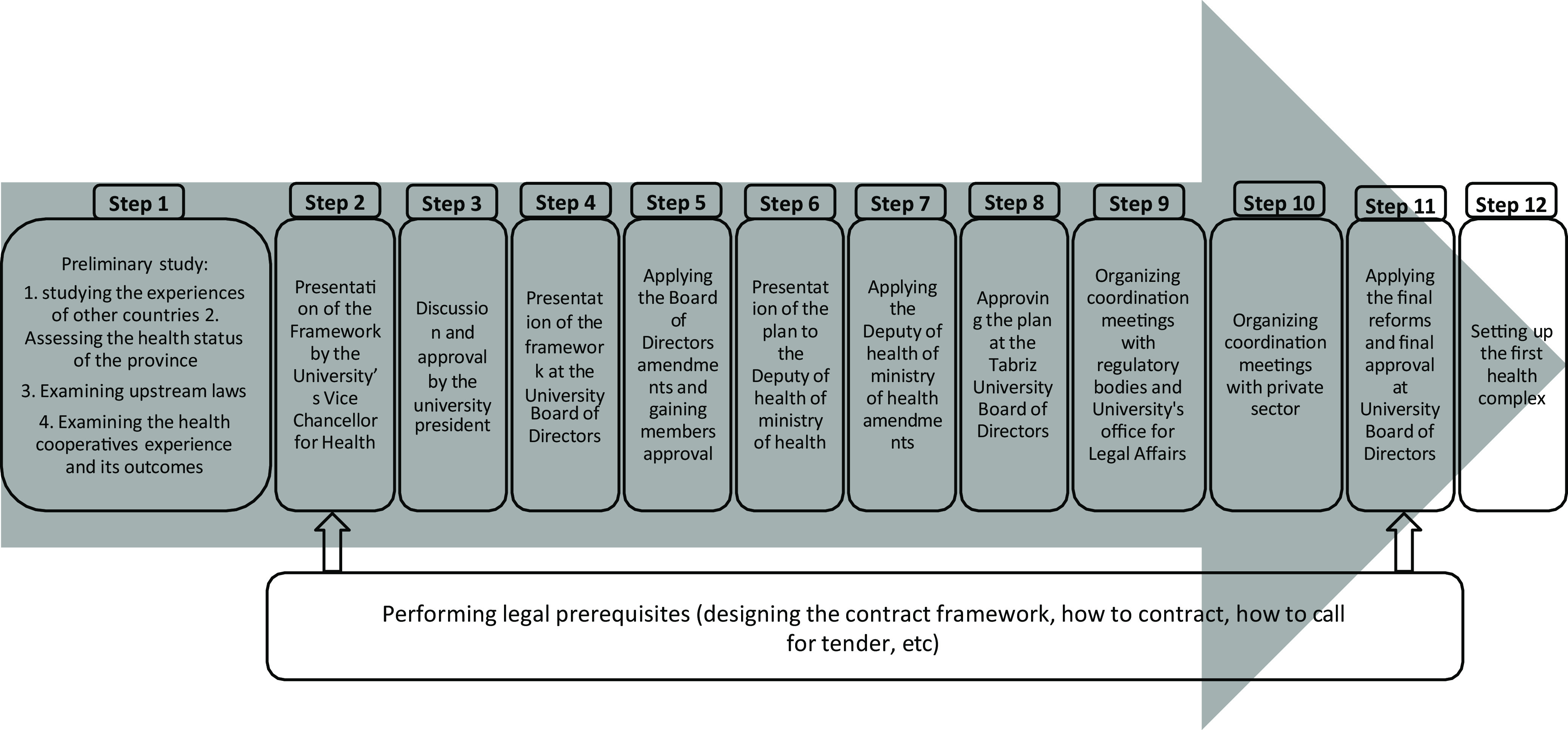
The process of designing health complexes in East Azerbaijan Province, Iran

The development and preparation of PPP policy in providing PHC services at EAP were carried out in several steps (Figure 2).

To review the successful experiences in the world, 14 countries’ experiences and their used structure were studied. One of the challenges was that the countries in the region or countries with similar socioeconomic status were not found to use their experiences. Also in Iran, apart from the experience of the health cooperatives, there was no other experience of PPP in PHC.

Also, at the stage of initial design of HCs, one of the challenges was the rules and policies of related public organization. To address this problem, upstream rules were reviewed and facilitating laws were used to appropriately design and formulate this policy. In this regard, participant No. 5 stated: “… many authorities and organizations have been obstructing, based on their own organization’s processes and policies. But we have many rules in the country that allow the private sector easily participate in most areas. No one knew these … we could use these rules to design PPP policy (in EAP) … we studied these rules and extracted the positive points that help us to justify our plan …”.

In the process of modifying the initial model by taking the direction of the university president and senior officials from the MOH, the current challenge was to reform the plan based on their views, which in the opinion of the policy designer team damaged the quality and design of the plan. To meet this challenge, the policy designer team explained the plan to the authorities as much as possible and get their feedback, otherwise they had to make changes. In this regard, participant No. 9 said: “… they accepted some of our programs and didn’t accept some others, and because they were our superiors we had to accept their opinion. We tried to make them to accept all the changes but it didn’t happen, they accepted some of the changes. The plan was implemented but incompletely, (anyway) it was better than nothing … .”

During the time taken to obtain approval from upstream managers, the design team did the legal process. For this purpose, joint committees were formed with the University’s Vice Chancellor for resource management affairs and issues such as contract format, contract method and call method were discussed and finalized. Joint meetings with the university’s legal affairs office were also held, and the plan was revised and approved using their comments.

At the next stage, representatives of the private sector were invited and the plan was presented to them during various sessions. Their views were reviewed and based on their comments initial framework changed somewhat. Insurance companies were also invited to participate in project coordination and finalization meetings along with the private sector. Provincial insurance representatives found the plan useful and positive, but country-level organization’ rules obstructed them from participating in the plan.

In the public sector, no one opposed the whole plan. There were only some disagreements from some departments that during the policy formulation the comments of managers and heads of the different departments were taken and based on their views, the necessary changes were made, and finally, agreement was reached. So, at the macro level, the Health Deputy of MOH and the Minister of Health agreed, and at the local level, MPs, religious authorities, nongovernmental organization (NGOs), municipalities and government organizations supported the plan. At the implementation stage, some individuals and departments disagreed with the details of the implementation. Some public sector employees opposed the plan because of fear of losing their job position and some private doctors because of the decline in private practice due to the construction of a HCs. Despite these oppositions not having a strong base to oppose the plan, the policy designer team tried to resolve it as far as possible using various methods such as bargaining, negotiation and joint meetings. More information on stakeholder analysis is provided in Gharaee *et al*., (2019).

One of the challenges in setting up of HCs was the shortage of eligible private companies to participate. For this reason, a number of private companies that have good performance in health cooperative plan have been asked to participate in this plan. But companies were reluctant to participate because of the negative experience they had with participating in the health cooperative project. To address this problem, representatives of private companies were invited to meetings in which various dimensions of the project were presented to them. Consequently, some of private companies announced their willingness to participate in the plan, which the HCs were assigned to them without bidding.

After the approval of plan by university’s board of directors, the first HC launched in June 2014. About 8 months later, following initial evaluations of the plan and carrying out some reforms, the other HCs began to work from February 2015. Then, from May 2015, the remaining cities of the province were gradually covered (Takian *et al*., 2015). HCs were used as a context for the implementation of PPP in EAP.

PPPs were launched in EAP with the public budget. Since the MoH was focused on implementing health evolution plan in the country and part of it emphasized on PPP, the idea of TUOMS was welcomed and substantial funding was committed to the PPP plan of EAP. District health networks pay capitation to the private HCs, and they should provide the physical space, human resources and other equipment.

If providing PHC services for residents of a defined region is transferred to a private HC, all public health centers in the region will cease to operate, and public and private HCs will not work in a region simultaneously. If there were public health centers in the region, its building would be rented to private companies. Otherwise, the contracting companies were required to rent a suitable location. In some cases, municipalities provided the required space. The district health network is responsible for verifying the suitability of the location.

Like the health cooperatives, all current costs of HCs, except for the paper forms, vaccines, some medicines and health equipment, were borne by the private company.

### HCs structure

HC is a health services center, which provides defined service package to defined population based on the given capitation within the framework of MOH and utilizes the abilities of public and private sectors (Table 1).

**Table 1.** The various constituent parts of health complexes and their duties

Health complex	Comprehensive health center	Management headquarters	In this part, director of HC, along with headquarters experts (diseases control, family and school, environmental and occupational health experts, etc.), is responsible for managing health programs, including planning, coordinating, training and empowering employees and people, and monitoring and evaluating queue units
		Referral clinic	Specialized medical services: This part has four main specialists including pediatrician, internist, gynecologist and psychiatrist, which provides outpatient care services to referred people with government tariffs. In case of direct referring to this center and without passing the referral path beginning from health centers and family physicians, visit costs of specialists should be paid with private sector tariffs Nutrition counseling: Responsible for counseling referral patients and sending feedback to health centers Mental counseling: Providing consultation for referral patients and sending feedback to health centers Quit addictions unit
		Laboratory	Laboratory services are provided at CHC or a place outside it, considering people access and after obtaining the required license from district health center and purchasing services from the public and private sectors
		Administrative and financial affairs	Responsible for administrative and financial affairs of HC
		Midwifery services	Responsible for providing midwifery services to referrals and sending feedback to health centers
	Health center	Family practice	Provides outpatient care services to the covered population. One of the family physicians working at the health center is responsible for the center. Outpatient care services during non-office hours and holidays and emergency services during 24 hours should be provided by physicians of the health centers at HCs locations to the covered population
		Family health nurse	Providing first-level service package including PHC services such as vaccination, maternal and child care, family planning, public education, environmental health, and to the individual, family and covered population
		Admission	Preferably associate or bachelor’s degree of medical records

**Figure 3.**
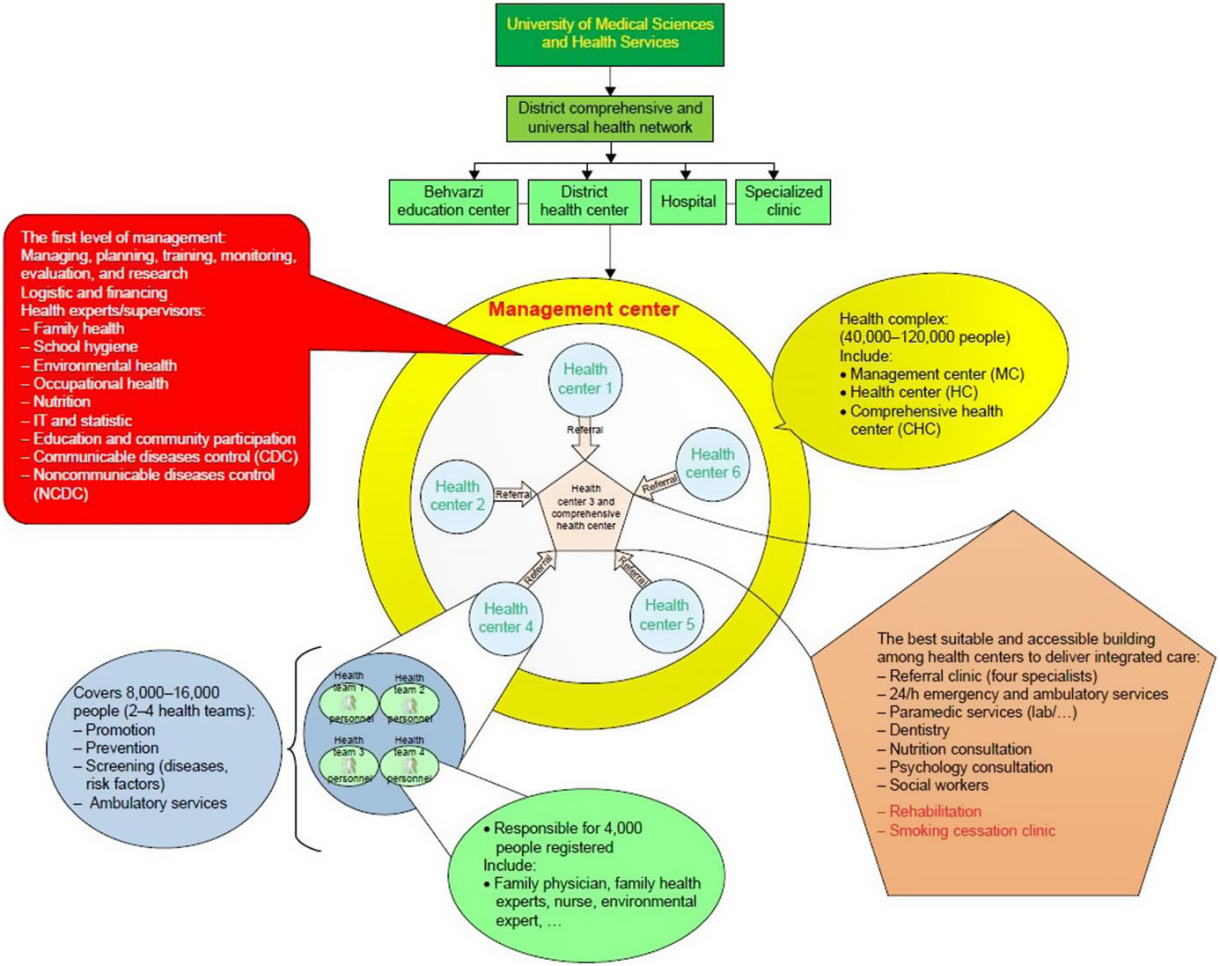
The health complex structure

Prior to the construction of HCs, the structure of PHC was completely governmental, as shown in Figure 1. After the construction of HCs, the structure of PHC delivery changed (Figure 3). HCs in the marginalized areas of the cities are assigned to private sector in the form of PPPs. Some of the province’s districts were also fully privatized, while others were half PPP and half governmental. Other districts in which there was no proper background (such as the lack of private company), PHC services are provided by public sector.

More information on HCs is provided in Tabrizi *et al*. (2016), Bakhtiari *et al*. (2017) and Tabrizi *et al*. (2019)

#### Contract content and its formation process

The policy design team from university’s vice chancellor for health formed joint teams and committees with the vice chancellor for resource management affairs and finalized the contract format, recall procedure, contracting process and other relevant issues. In the next step, joint meetings were held with the university’s legal affairs office and the primary plan was reformed using their comments. Then, several meetings were held with representatives of the private sector (have experience in the health cooperatives in 1998, and their good reputation had been proven) and comments were taken, and eventually some changes were made to the primary plan, using their comments. Again, in some meetings, with the presence of representative of university’s legal affairs and vice chancellor for resource management affairs, the primary contract was finalized and approved by university’s legal affairs office. At the final step, final format of the contract was submitted to the university board of directors and after minor amendments, the contract format was approved by university board of directors. The process of assigning HCs and contracting with the private sector has been elaborated in detail in the study of Bakhtiari *et al*. (2017).

The contract between the public and private sectors consists of an introduction and 14 articles that signed between district health network and private companies (Table 2).

**Table 2.** Contracts for the assignment of primary health care to nongovernmental sector

Article	Subject
Contract subject	Region health management and provision of healthcare services according to the laws and regulations of the MOH, which the universities of medical sciences are required to implement
Duration of contract	All contracts are adjusted for a period of 3 years and after that, in case of agreement and satisfaction of performance, they can be extended or moderated. The deployment of human resources and the launch of services provision that must be verified by the contractor are the basis for the start of a contract and capitation payment. Guaranteeing the contract is annual and based on the capitation of same year
The covered population	The census and blocked population is the basis for payment for the first quarter of the contract. During implementation of the contract, population changes can be calculated quarterly according to the information recorded in the electronic system that will be the basis for payment
Place of providing services	CHCs (management headquarters of health complexes) and health centers
Types of services	(A) Services included in capitation such as vaccination, basic and periodic visits, target groups care, oral and dental health services for target groups and pre-pregnancy tests for mothers are free and no franchise is gotten from the covered population for providing these services. (B) In case of services not included in capitation such as outpatient visits and pharmaceutical and para-clinical services, franchise is gotten from the covered population for providing these services and the remaining costs are received from the insurance companies. (C) About services which not included in capitation such as injections and dressings, and some dental services, the total cost received from service receivers or insurance companies under government tariffs
Monetary value of the contract and financial turnover	The service capitation is based on the announced capitation by the TUOMS and the mutual agreement. Total cash and noncash incomes of services provision not included in capitation deposit into the bank account of the network and after taking legal procedure, will be returned to HC account in full. Review and confirm the covered population in the information system done by the employer quarterly that is the basis for reimbursement to the contractor for the next quarter
Contractor’s obligations	Work hours, how to deal with the breach of contract, to record information and legal fractions
Employer’s obligations	Provide required hygienic items, conduct monitoring and evaluation, reimbursement conditions and education
Monitoring and evaluation	Monitoring and evaluation content, bonus and its payment method (annexed)
Performance and obligations Guarantee	The contractor must submit a valid bank guarantee equivalent to 10% of the total amount of the contract to the employer when signing the contract. If the contractor fails to comply with any of his obligations, the employer will confiscate the guarantee without any legal formalities in favor of the university
Government employees intervention prohibition act	The contractor is not subject to intervention prohibition act and if it is found otherwise, the contractor is obliged to compensate the employer for damages
Termination of contract	Employer can terminate the contract unilaterally and terminate the activity of contractor if finding any contractor’s violation of the provisions of this contract and the contractor has no right to protest and in case of damage from the contractor, the employer can confiscate the guarantee and financial claims of the contractor and also claims any damages occurred more than the amount of guarantee and claims
Dispute resolution	The parties will try to resolve disputes arising from the contract in a friendly manner, otherwise the matter will be resolved in the presence of representatives of vice chancellor for health and contractor at the Financial and Trading Expert Commission of the University
Residence of parties to the contract	Each party to the contract is obligated to inform other party in writing if the address is changed; otherwise, the previous address is valid for official notification

#### Service package

HCs are responsible for providing or managing the service package at two levels (Table 3).

**Table 3.** Health complex service package at first and second level

Service package at first level	Regional health management	Identifying the geographical environment, identifying the covered region in terms of population, residential places, workshops, etc., identifying the health problems and difficulties in the region, health information management, attracting public participation, monitoring and evaluation of health team services, etc.
	People and employees empowerment services	Public education and the promotion of healthy lifestyles, implementation of national and provincial programs, attracting public participation, inter-sectorial coordination in health, forming support groups, training and continuous empowerment of service providers
	Provide health services in accordance with the plans of the Ministry of Health and University	Maternal care in reproductive age, infant and children under 6 years care, nutrition health and safety in accordance with the MOH plans
	Referral of clients requires additional services to higher levels	Having a work plan for specialist of referral clinic, recording of referral to higher levels in the information health system, coordinating with higher levels for patients’ referral through the system and set a visit appointment, tracking feedback from the referral receiver, setting and submitting the required reports
	Social activities	Informing about the plan, forming a public board of trustees with participation of authorities and influential people of the region, using health volunteers as a bridge between health centers and people, creating information campaigns and cooperating with external organizations on social activities such as poverty reduction, campaigns for quitting addiction, reducing traffic accidents and increasing physical activities
Service package at second level	Health services management	Cooperation with health centers in planning to improve the health of covered population, helping to improve health and self-care programs, recording and categorizing chronic and important diseases in the region and planning to control them
	Care services	Filing, treatment and monitoring according to clinical guidelines, giving feedback, referral to other specialists if needed, tracking patients at other levels, empowering people in accordance with family physicians, tracking hospitalized patients, guidance and continuous education for family physicians
	Management services	Monitoring the referrals of family physicians, summarizing and analyzing the required indexes and providing the relevant reports, coordinating and training sessions with health center staffs, contributing to develop and update guidelines for providing educational and research services in accordance with the TUOMS programs

#### Human resources

Human resources were recruited by private companies, among those who are graduated from Medical Sciences Universities and qualified in accordance with specifications announced by the district health network. To start working as a healthcare provider, volunteers should pass an initial training course and get a certificate approved by the health network. These training courses are run by the health networks according to standards and methods approved by the vice chancellor for health.

During these courses, experts at the Provincial Health Center and the Health Network educate various MOH’s guidelines for providing PHC services (including maternal and child care, pregnancy care, elderly care, vaccination, disease prevention and control, monitoring and evaluation, monthly reports, etc.) in the form of training sessions that last for 1 month. At the end of the course, participants will take the end-of-course exam and, if they pass the exam successfully, are allowed to start working in private HCs in marginalized areas, and if they fail the test, they will have to take the course again. All the above-mentioned procedures carried out by the public party, the health network and private companies are not involved in the process of conducting classes, testing and licensing.

Governmental staff who worked in public health centers in marginalized areas, before the implementation of PPP plan, after assigning these centers to private companies, are transferred to the public HCs in downtown to compensate its possible low human resources. Some employees who have more experience and better performance work for a period of time (at least two months) in the private HCs along with staff newly recruited by private sector. So that in the first month, public sector’s employees provide services and private sector’s employees watch. Then, during the second month, private sector’s staff provide services under the supervision of public sector staff; if an error exists, public sector staff will fix it. This process continues until private sector employees can provide services independently. Afterwards, private staff like public staff participate in in-service training classes and courses organized by the health networks with the aim of empowering staff.

Private HCs cannot fire or replace staff repeatedly unless providing justified reasons and after confirmation of health networks (more details in Bakhtiari *et al*. (2017)).

#### Monitoring and evaluation

Before the launch of the private sector work in the form of HCs, monitoring and evaluation checklists were designed according to the service package and contract. After designing the monitoring and evaluation package, private sector was asked to read the package and give their comments. Private sector’s comments were taken and necessary reforms were made. Payments to HCs are based on monitoring and evaluation results done by health networks. Eighty percent of the contract is paid monthly and the remaining 20% paid once every 3 months based on the results of monitoring and evaluation (Table 4) (more details in Tabrizi *et al*. (2019) and Bakhtiari *et al*,. (2017)).

**Table 4.** Monitoring and evaluation and payment methods to health complexes

Monitoring and evaluation	Self-assessment	Service providers evaluate their own performance monthly
	Evaluation of health complex headquarters	HCs headquarters evaluate the health centers every 2 months (besides, HC headquarter experts asked questions about service delivery process from the covered population)
	District headquarters evaluation	Headquarters of district health center evaluate HC and health centers once every 3 months by a checklist similar to the previous steps (reimbursement basis to HCs)
	Assessment of the province health center	Annual evaluation of the health network, district health center, and HCs and health centers by the vice chancellor of health
Reimbursement	Capitation	• The amount of capitation varies depending on the type of services assigned to HC
		• May change every year or once every few years
		• The provision of services included in the capitation is free and no franchise is gotten for delivery
	Fee for services	• Cost of services not included in the capitation is received according to government tariffs and is equal to current roles of the country
		• Customers outside the referral system must pay 100% cost of specialized services according to private sector tariffs
	Case payment	In the case of doing specific actions and providing special services (diagnosis of rare, contagious and life-threatening diseases, etc.)
	Incentive payment (BONUS)	Based on the results of the evaluation and getting a score more than 90, per score, 1% of capitation will be added to the total monetary value of the contract
	Pay for quality mechanism	After allocating funds from the vice chancellor for health to districts health centers, payments to HCs and health centers were performed on the basis of the performance and quality of the provided services. Payment to employees is based on the predefined formula with a specific ceiling (defined as a percentage of the capitation). This payment method has been implemented in the public sector and will be implemented in private centers and HCs, at the next stage

#### Referral system

**Figure 4.**
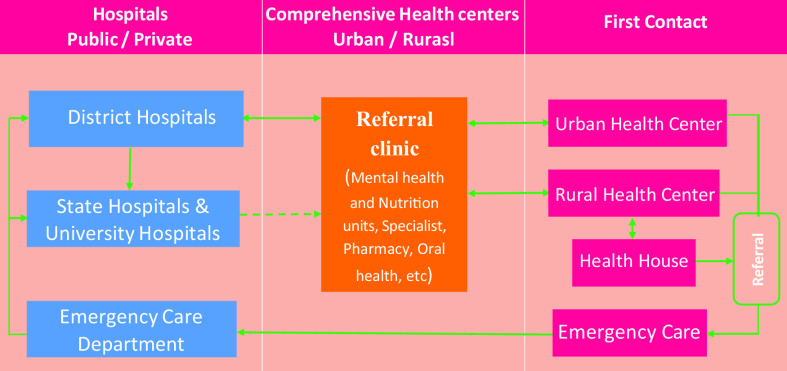
Referral system in health complexes

Referral system is a set of processes determining person’s movement and communication path through triple levels of health services provision. Feedback of the provided services along the referral path is recorded in the individual health electronic file to inform the family physician (Figure 4).

#### Health socialization

In health system reforms, one of the concerns of policymakers is to attract public participation and cultivate public culture in order to institutionalize reforms. In this regard, PPP designers in EAP had some plans, including notification about the plan to overcome cultural problems, forming public boards of trustees with participation of people’s representatives, region’s officials and influential individuals to identify, prioritize and solve regional health problems, establishing communication between people and institutions with HCs and following the issues related to public health, applying health volunteers as the bridge between the HC and its subsets with people, creating notification campaigns in marginalized areas aimed at promoting public health literacy such as campaigns to reduce salt intake, reduce fat intake, reduce sugar intake, increase consumption of dairy products, control anger and stress, multi-sectorial cooperation with other organizations and institutes in the field of social activities such as poverty reduction, campaigns to quit drug addiction, reduce traffic accidents, increase physical activity through creating sport spaces and holding public exercise programs.

#### Outcomes and achievements of the plan

**Figure 5.**
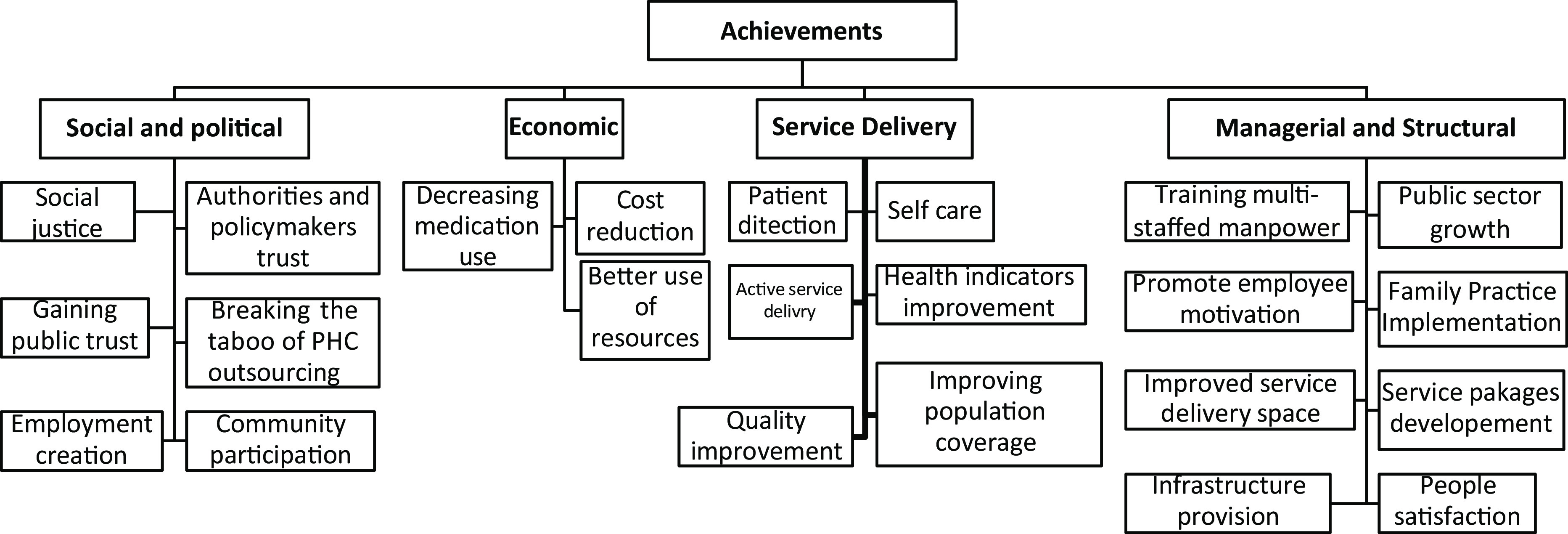
Results and outcomes of the public–private partnership initiative in primary health care

The outcomes and achievements of PPP in providing PHC announced by interviewees were categorized into 4 main themes and 23 subthemes (Figure 5).

According to interviewees’ opinions, the most important achievement of designing and implementing this plan is social justice promotion to which all the participants agreed. Also, despite its weaknesses and difficulties, interviewees considered this initiative essential to reform PHC system. On the other hand, almost all the participants considered the private sector more successful than the public sector in PHC. They believed that the performance of private HCs in areas such as responsiveness, quality, cost-effectiveness and other items was better than the public HCs.

As part of this plan, 18 private HCs and 17 public HCs were set up and 18 contracts were signed with private companies. On the other hand, 1100 people were recruited, all of whom after passing initial training course gained license to work in private HCs and its subsets as family health nurses. All HCs’ managers and headquarter staff, working in complexes, attended health management training courses.

#### Trends of some of key indexes before and after PPP

**Figure 6.**
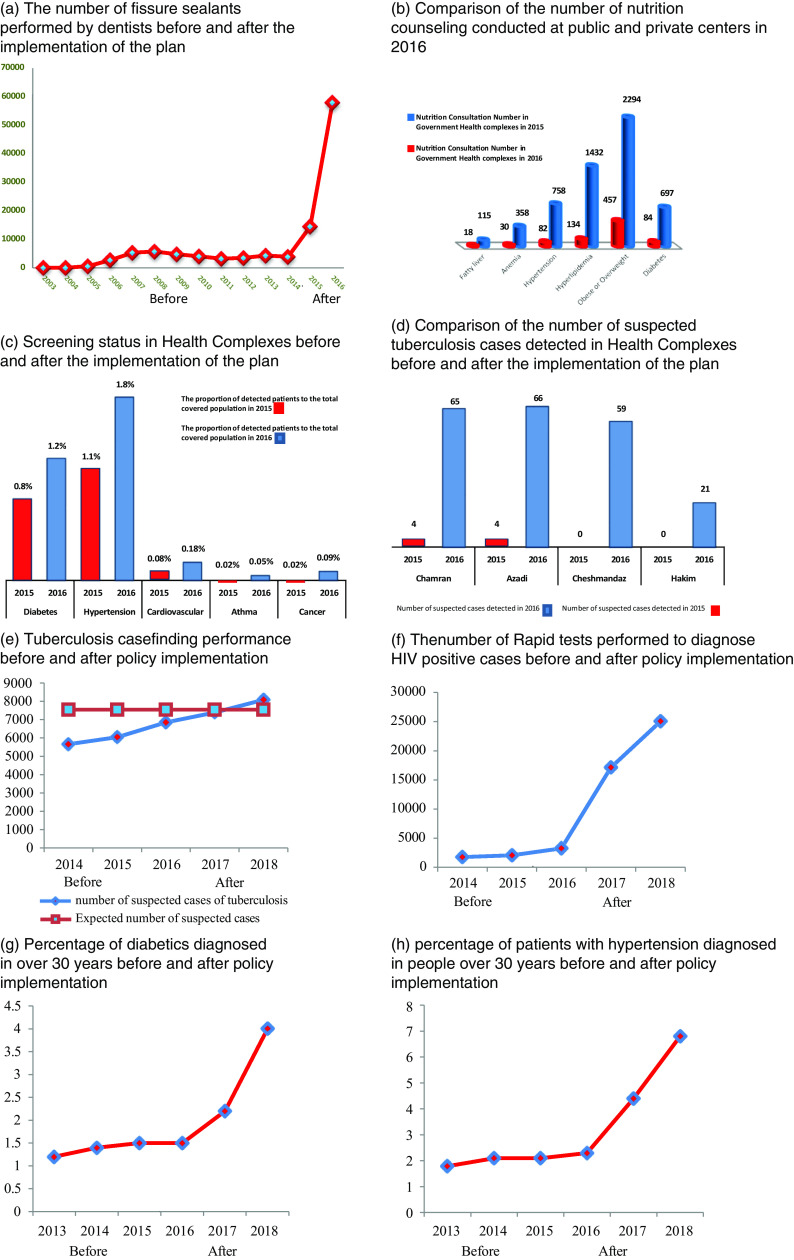
Health indicators before and after implementation of public–private partnership in primary healthcare policy

In Figure 6, a few quantitative health indexes, indicating the performance of HCs before and after the implementation of PPP plan, are given. Results of the total extracted health indicators are published in other articles.

Figure 6a indicates that the number of fissure sealants increased dramatically after the implementation of the PPP plan. The increased number of nutrition counseling in different diseases after the policy implementation is indicated in Digure 6b. Since implementing PPP plan led to providing active services, as shown in Figure 6c, screening of different diseases has increased. Figure 6d shows the changes in number of suspected tuberculosis cases detected, in four private HCs in marginalized region, 1 year after implementation of PPP plan. Figure 6e shows that after policy implementation the number of tuberculosis case finding in proportion of expected number, which shows the performance in case finding, has increased after PPP plan. According to Figure 6f, the number of rapid tests to diagnose HIV-positive cases has improved in comparison with before the policy implementation. Eventually, Figure 6g and 6h shows the significant increase in percentage of diabetes and hypertension diagnosed in people of over 30 years after policy implementation.

#### Challenges of the plan

In view of interviewee, some challenges that were in the design and implementation of PPP in providing PHC are provided in 5 themes and 14 subthemes (Table 5).

**Table 5.** Challenges of public–private partnership in primary healthcare policy in East Azerbaijan Province, Iran

Main theme	Subtheme	Direct quote of participants
Political	Unstable support of authorities	Participant No. 2: “We trusted the authorities … but then they didn’t stand on their promises and left us behind to preserve their post … my personal impression is that we had relied on some absurd promises that later hit us… like providing enough and on time budget…”.
Infrastructural	Lack of scientific evidence	Participant No. 7: “Our biggest challenge was not to have the same experience in our own country or in the countries of the region that were similar to us, to use their experiences”.
	Lack of referral system	Participant No. 6: “…Necessary coordination with level two (second level of health services) was not achieved. For example efforts, that should be made in order to the vice chancellor for Treatment affairs and Specialty Clinics accompanied us so we can set up a referral system, was not done…”.
	Rules and Regulations	Participant No. 5: “…Somewhere there were no rules and somewhere they (rules) were obstacle, that we trying to solve them in some ways, and sometimes they were helpful … in general the rules seemed to me to be a challenge…”.
	The shortage and inability of private companies	Participant No. 4: “…Another problem we had was the shortage or lack of companies and people to do this. Well, there were a lot of volunteers in clinical sector, but the one who wanted to participate in primary health care was very little…”.
		Participant No. 6: “…Private companies and groups that volunteer for partnerships lack the necessary technical, financial, and managerial capabilities, and they cannot provide us with what we want. …”.
	Shortage of physician	Participant No. 3: “…We had one physician appointed for every 4,000 people … Now some centers have contracted one physician for every 12,000 people to compensate for the shortage of physicians in this way, which has led to services not to be provide a as we expected…”.
	Information system	Participant No. 3: “…Another challenge was that the information system that was created was not perfect and did not meet our needs and lead to some problems…”.
Cultural	Lack of people participation	Participant No. 10: “The next challenge we have is the lack of people’s participation. Suppose you live in a city of over 20,000 and you have a cold right now … You can easily refer to a lung specialist… a signature white checkbook have given to you… you say I don’t need primary health care and I go straight to a specialist whenever needed…”
		Participant No. 8: “People lack the culture and readiness to believe in the first level, which the first level can provide them with valuable services by family health nurse and general practitioner and can meet most of their needs”.
	Lack of common language	Participant No. 7: “…Many of our problems stemmed from the fact that we didn’t reach the common language, which was something I always felt…”.
	Negative experience of health cooperatives	Participant No. 1: “…We invited three health cooperatives (private companies) who worked with us in 1998 (health cooperative plan). Once they had been defeated, they had been lost at that time (stopping the plan).
		Therefore, they couldn’t trust in current plan (Health Complex) and start with us again…”.
	Lack of trust in private sector	Participant No. 1: “We still do not believe in the private sector … the public sector mainly looks at the private sector as a competitor…”.
Between-sectorial	Non-cooperation of insurance companies	Participant No. 2: “…There were areas where insurance could be very effective and help the policy implementation, if they cooperate, but unfortunately they didn’t … one of the major challenges with this plan is that we couldn’t engage the insurance companies”.
		Participant No. 3: “Insurer companies are those that do not have decentralization and are still waiting for the order from the center (top level of organization). That is, in the provinces nothing can be done unless their headquarters in Tehran (Capital of Iran) decide and give permission…”.
Economic	The country’s economic problems	Participant No. 9: “…it could be said that situation was very good at the start, but over time the economic situation in all areas, not just health, got poor, this influenced the implementation of the plan…”.
	Unsustainable financing	Participant No. 11: “Unsustainable financing and that we don’t know when and how much we are going to get, lead to that we can’t planning our work as it should be…”.

#### Weaknesses of the plan

Weaknesses of PPP in providing PHC plan in view of interviewees are provided in 3 themes and 8 subthemes (Table 6).

**Table 6.** Weaknesses of public–private partnership in primary healthcare policy in East Azerbaijan Province, Iran

Main theme	Subtheme	Direct quote of participants
Lack of infrastructure	Lack of background	Participant No. 6: “….The implementation of the policy took place very quickly. Accuracy was sacrificed for speed…. We should have wrote a 10-year plan, made culture, trained manpower, implemented exact census, involved all companies and organizations, and in fact, have developed an all-encompassing development.”
	Lack of notification about the plan	Participant No. 3: ” I think they (designer team) did not make the importance of the policy as transparent as needed; they should have attracted the statesmen, policymakers, and society.”
	Neglecting private sector physicians	Participant No. 11: “We did not involve the private sector physicians in this plan. If we implement this plan properly in an area, then what should do the physician working in private clinic in that area? Dies of hunger? So there will be resistance in the community….”
	Lack of a pilot study	Participant No. 11: “…They rushed to implement the plan… Implementation of the pilot study could help identify and remedy the project’s weaknesses and even some challenges…”.
Monitoring and evaluation	Failure to define correct goals and indexes	Participant No. 3: ” For example, they should have said that we ask you to control this percentage of pregnant mothers or if, there is one case of a specific disease, we will fine you, or we will award you if you can control the blood pressure of this percentage of patients; in this way, I would follow the patients and the people…”
	Long monitoring and evaluation process	Participant No. 4: “Monitoring and evaluation lasts for a month; then the score will be given one month later…”
	Insufficient transparency of checklists	Participant No. 7: “…different individuals had different perceptions of the checklist, it means that the grade I evaluated was different from a different level expert’s grade…”
Contract	Short-term contract	Participant No. 8: “the contracts period should not be one year, they must be contracted for at least 3-5 years…”
	Structure of pay for performance	Participants believed that the part of employees’ salary that is based on performance is low and not enough to influence their performance or behavior. In this regard, participant No. 3 stated: “These percentage (of salary) should be changed so that we can influence employee behavior.”

## Discussion

In recent years, many countries have contracted NGOs to provide health services, as a mean to deal with a lack of motivation, as well as deficiencies in the coverage, quality and efficiency of healthcare system (Greve and Schattan Ruas Pereira Coelho, 2017). The aim of this study was to introduce the experience of applying PPP in providing PHC services in EAP, Iran.

Based on the results, social justice was the main goal of policy implementation. It seems that the coincidence of this policy’s implementation with the health evolution plan brought political support and considerable public financial resources for this plan. In general, most participants believed that because of the technical expertise and experience of policy designer team, availability of financial resources and political support, this plan was designed and implemented.

The main achievement of this policy, in view of interviewees, was the improvement of social justice (through the improvement of access, quantity and quality of service). The interviewees believed that the PPP plan had improved the access and utilization of poor and marginalized people. In the study conducted by Bakhtiari *et al*. (2017) exploring the results of PPP in PHC policy at EAP, it was shown that financial, physical and even cultural access (service acceptance) to PHC services has been improved (Bakhtiari *et al*., 2017). The results of the study carried out by *Reeve et al*. (2015) showed that, about 6 years after the strengthening of PHC in Australia, the quantity and quality of care provided to marginalized people have been dramatically increased, which was higher in deprived areas (Reeve *et al*., 2015).

Other achievements were active service delivery, providing secondary health services alongside PHC (service package development), changing the role of the public sector, people satisfaction, etc. Based on the results of Dehnavieh *et al*. (2018) study, decentralized planning, strengthening the engagement of the private sector, using the performance assessment methods more appropriately, using the prospective payment method, strengthening the referral system, strengthening service continuity, facilitating financial access and increasing geographical access especially in marginalized areas are the strengths of this policy (Dehnavieh *et al*., 2018).

Loevinsohn and Harding (2005) believe that in developing countries, assigning health services to nongovernmental providers leads to better results than governmental providing services by public providers (Islam *et al*., 2018). The results of this study showed that after implementing PPP plan in PHC system of EAP, many health indexes have improved. In 2000s, a major change took place in Brazil health system, and for the first time, the management of a PHC center was assigned to an NGO in the form of an agreement. Analyzing the results of this plan, in the form of some indexes, shows that outsourcing the provision of PHC services increased the number of referring to PHC provider centers on average once a year per person in the state of Sao Paulo in Brazil and also because of the implementation of this strategy, hospitalization due to preventable diseases has declined (Greve and Schattan Ruas Pereira Coelho, 2017).

The implementation of reforms, programs and new plans in health system has always faced some problems and challenges. In formulating and implementing PPP in providing PHC in EAP, some challenges have been raised.

The biggest challenge facing the implementation of the plan was economic barriers. At the beginning of the plan implementation, the budget was timely provided, but after the government faced economic problems, providing financial resources was discontinued and there were serious challenges in the continuation of the plan. This issue has also been highlighted in the study of Maluka (2018) conducted in Tanzania. The results of the mentioned study showed that shortages of financial resources and delays in payments have endangered the continuation of PPP plan (Maluka, 2018).

The other challenge was cooperation of health insurance companies. According to interviewees’ opinions, if insurance companies get involved in this plan, it can contribute greatly to the removal of economic barriers, but this has not happened for various reasons, including centralized structure of these companies.

Other challenges include rules and regulations, weak participation of people, lack of physicians, negative experience caused by the cessation of health cooperatives plans and weak information systems. Lack of common language and lack of trust between private and public sectors are also considered as a challenge. It seems that in addition to payment mechanisms, contracts must clearly outline the expectations of both parties. It is necessary to have ongoing coordinating meetings between the public sector, service providers and other stakeholders for building the trust and meeting the expectations of both parties.

Shortage and disability of private companies to participate were another challenges of this plan. Policy designer team tried to solve it by assigning the first HCs to known companies without bidding. Given the examples of the relatively successful use of NGOs as contractors in different countries such as Brazil (Greve and Schattan Ruas Pereira Coelho, 2017), Guatemala (Cristia *et al*., 2011) and Tanzania (Maluka, 2018) and since one of the challenges posed in PPP for providing PHC policy in EAP was a shortage of private volunteer companies and sometimes their inability to provide preconditions of public sector, the use of NGOs can pave the way for solving this challenge.

In this study, most consensus was on the lack of infrastructure (such as designing mechanisms for collecting information to determine the success rate of the plan, lack of implementation of the plan in the form of pilot study, weak notification on the plan, lack of attention to physicians who work in private sector) which is the main weakness of the PPP plan. In view of participants, some problems and weaknesses were predictable and some of them not predictable and have been identified in the process of implementation. But it seems that, as many interviewees said, conducting a pilot study could have been contributed to identify and fix these weaknesses.

Despite the mentioned achievements and weaknesses, stakeholders have described this policy relatively successful and helpful in solving the problems of the PHC system. This seems to be a good ground for gaining stakeholders’ support to form a powerful coalition to address problems.

### Application in Low and Middle Income Countries

Recent experience has shown that PPP’s success in health sector is largely influenced by its design and the context in which it is implemented. To overcome this, PPP models need to be adapted to the conditions of each country or context. The PHC system in Iran is different from other countries. The achievements in Iran’s PHC have led to expectations for this system going up. With the implementation of the Health Network System, this system has focused on issues such as maternal and child health and infectious diseases that have been significantly successful and have improved these indicators. But over time, the landscape of people’s illnesses and health problems has changed. For example, chronic illnesses and mental health problems have increased. But the structure of the PHC system has not changed in accordance with these changes. Health networks were successful mostly in the rural but did not perform well in the urban areas. Now urban areas need to be addressed and in this regard, PPP plan in providing PHC services in EAP is also focused on urban areas.

The experiences presented in this study can be helpful for countries planning to design and implement PPP in providing PHC services. Also, this study provides an insight about the perception of different stakeholders on PPP in providing PHC services that can be useful for low-income countries. One of the challenges of PPP plans, especially in developing countries, is the lack of rich information to judge whether or not plans are successful. To overcome this, it is better to define some indexes and standards for determining the success rate of the plan and design the mechanisms required to collect the relevant data, or even include it in the contract as one of the private sector’s duties. On the other hand, good design and implementation of a PPP plan do not guarantee its success, but the success of such plans requires continuous monitoring and evaluation to identify possible barriers and challenges as well as facilitating factors. Due to political unsustainability that sometimes lies in developing countries and also was among the problems of implementing the plan in EAP, it seems that applying civil pressures is critical to prevent retreating from reforms. For applying civil pressures, attracting the support of people and stakeholders seems to be essential. In this regard, media play an important role in reflecting successes and achievements of plans. Power of media can also be used to minimize the resistance and motivate various stakeholders. One of the most important requirements for implementing PPP plans in developing countries is creating management capability in public sector for monitoring and evaluating the performance of private sector. This requires the development of proper indexes and objectives, establishment of a targeted and accurate monitoring and evaluation program, development of high precision tools and finally pay for performance based on the results of monitoring and evaluations. One of the necessary and useful tools in this area is implementation and use of information systems that can help improve the accuracy and precision of monitoring and evaluation and payment based on it. Based on developing such a structure, specialized and independent private companies can be used to measure and improve quality.

## Recommendations

The results show that while this plan is regarded as an essential and effective way to provide social justice, implement family practice plan and achieve UHC, challenges such as political and financial unsustainability, delay in reimbursements, lack of stakeholder co-operation and lack of continuous quality of services evaluation have had negative implications for the plan which could jeopardize its existence. Overcoming challenges, more success and expanding this plan require accepting the private sector as one of the main sources of the health system for achieving the UHC and require more support from authorities of TUOMS, MOH, provincial government, insurance companies and other provincial and national institutions. It is recommended that custodians in the vice chancellor for health and TUOMS adopt strategies in order to ensure adequate funding and its timely payment. One of the solutions is attracting the cooperation of insurance companies. On the other hand, successful formulation and start-up of the plan do not guarantee its success, so a mechanism should be designed through which monitoring the plan can be continued at the next steps to resolve its possible challenges and weaknesses. The next proposal involves holding coordination meetings to involve stakeholders in the process of managing and organizing the implementation of the plan so that a common language is created among all stakeholders. Finally, it is suggested to inform the public through the media to increase public participation in order to the plan becomes a public demand, and in result politicians and institutions have a greater tendency to support the plan.

It should be noted that the experience gained from the development and implementation of this plan can be useful in the implementation of PPP in other health areas as well as in nonhealth areas. On the other hand, the experience gained from the implementation of this plan can be helpful for other countries with similar economic, political and social conditions.

## Study limitations

In part of this study, the existing documents were used to extract data. Given these documents were not developed for research purposes, they had less proportionality with study objectives. Also, due to the weakness of electronic information systems in recovery of some information, changing the process of information recording in some health programs and the newness of some health programs, it was not possible for researchers to access some information.

## Conclusion

This study introduces the PPP plan in providing PHC in EAP which is a new experience and a new reform in this field that have brought considerable results and achievements. Improving access to PHC, especially for marginalized people, promoting population coverage, improving the quality of services, satisfying people and gaining the trust of authorities and policymakers are some achievements of this plan. The present study results show that if sustainable resources, trust in the private sector, sustainable political support and the support by all organizations and stakeholders, particularly health insurance companies, this plan can continue more successfully and lead to the improvement of the service comprehensiveness, population coverage and health indexes and finally lead to reduction of health costs.
